# Water Extract of Deer Bones Activates Macrophages and Alleviates Neutropenia

**DOI:** 10.1155/2013/617302

**Published:** 2013-06-09

**Authors:** Han-Seok Choi, Soon Re Kim, Se Hyang Hong, Jin Mo Ku, Min Kyoung Kim, Hye Sook Seo, Sung-Gook Cho, Sangtae Shin, Yong Cheol Shin, Seong-Gyu Ko

**Affiliations:** Department of Preventive Medicine, College of Korean Medicine, Kyung Hee University, Seoul 130-701, Republic of Korea

## Abstract

Extracts from deer bones, called nok-gol in Korean, have long been used to invigorate Qi. While neutropenia is not well detected in normal physiological condition, it could be a cause of severe problems to develop diseases such as infectious and cancerous diseases. Thus, a prevention of neutropenia in normal physiology and pathophysiological states is important for maintaining Qi and preventing disease progress. In cell biological aspects, activated macrophages are known to prevent neutropenia. In this study, we demonstrate that water extract of deer bone (herein, NG) prevents neutropenia by activating macrophages. In mouse neutropenia model system *in vivo* where ICR mice were treated with cyclophosphamide to immunosuppress, an oral administration of NG altered the number of blood cells including lymphocytes, neutrophils, basophils, and eosinophils. This *in vivo* effect of NG was relevant to that of granulocyte colony stimulating factor (G-CSF) that was known to improve neutropenia. Our *in vitro* studies further showed that NG treatment increased intracellular reactive oxygen species (ROS) and promoted macrophagic differentiation of mouse monocytic Raw264.7 cells in a dose-dependent manner. In addition, NG enhanced nitric oxide (NO) synthesis and secretions of cytokines including IL-6 and TNF-**α**. Consistently, NG treatment induced phosphorylation of ERK, JNK, IKK, I**κ**B**α**, and NF-**κ**B in Raw264.7 cells. Thus, our data suggest that NG is helpful for alleviating neutropenia.

## 1. Introduction 

Neutropenia is characterized by a low neutrophil number, which could be a risk of disease development including infectious and cancerous diseases [1–6]. Thus, therapeutic approaches such as administration with G-CSF or GM-CSF have been applied to prevent neutropenic phenomenon, while no trials are completely worked yet [7–10]. Macrophages, one of the players in the innate immune system, modulate immune response through inflammatory cytokines and reactive oxygen species (ROS) [11–13]. In neutropenic situation, macrophages could not be involved in inflammation because neutrophil depletion results in no reaction of monocytes and macrophages [14]. As macrophages produce CSF, an activation of macrophages is crucial for preventing neutropenia and secondary diseases [15–17]. 

Extracts from deer bones, called nok-gol in Korean, have been traditionally used as a drug invigorating Qi. Recent researches further reported various therapeutic effects of this traditional medicine on inflammatory disease [18, 19], bone resorption [20], and aging [21]. Furthermore, extracts of deer bones affected macrophagic activation in *S*. *aureus*-infected mice [22]. In this study, we demonstrate that water extract of deer bones (hereafter, NG) alleviates neutropenia through activating macrophages. 

## 2. Materials and Methods

### 2.1. Cell Culture and Deer Bone Extract Preparation

 Raw264.7 mouse monocytic/macrophagic cells were grown in Dulbecco's Modified Eagle's Medium (DMEM) supplemented with 10% heat-inactivated fetal bovine serum (FBS) and 1% antibiotics at 37°C in a 5% CO_2_ humidified incubator. Water extract of deer bones (NG) was provided by Nongshim Corporation (Republic of Korea). More information on NG production processes could be requested from the company. 

### 2.2. **In Vivo** Studies

Six-week-old male ICR mice were purchased from Orient (Sungnam, Republic of Korea). All procedures were performed on the approval of the animal care center of Kyung Hee University (Approval no. KHUASP (SE)-12-042). To induce neutropenia, mice were intraperitoneally injected with 150 mg/kg of cyclophosphamide twice in one week and then treated with cyclophosphamide (100 mg/kg) two times in another week. Mice were rested for one week with no treatment when treatments were switched. In addition, mice were randomized into 4 groups (normal, control, G-CSF, and NG; five mice per group) during resting time points. G-CSF (1 *μ*g/kg) or NG (200 mg/kg) was orally administered every day for the last procedure. At the end of the experiment, mice were sacrificed by CO_2_ inhalation, and cardiac blood was collected. 

### 2.3. Cell Surface Observation

Cells seeded into 60 mm culture dish at a density of 3 × 10^5^ cells/dish were treated with NG (25, 250, 500 *μ*g/mL) for 24 h. Cell surfaces were observed by taking images using a camera (Olympus, Japan) connected to a light microscope.

### 2.4. Cell Viability Assay

Cell viability was determined using WST assay (Dogen, Republic of Korea). RAW264.7 cells (1 × 10^4^ cells/well) were seeded into 96-well plates and incubated overnight. Cells were then treated with different concentrations of NG and incubated for another 24 hours. 10 *μ*L of WST solution was added to 100 *μ*L cell culture medium, and plates were incubated for 2 hours. Optical density (OD) was determined at 450 nm using a microplate reader (Versa Max, Molecular Devices, CA, USA).

### 2.5. Intracellular ROS Level Measurement

Raw264.7 cell pellets were washed with PBS and incubated with 20 *μ*M DCFH-DA for 1 hour at 37°C in the dark. After washing with PBS, green fluorescence (480 nm excitation/530 nm emission) was measured by flow cytometry (BD FACSCalibur).

### 2.6. Detection of Nitric Oxide (NO)

NO production was measured by adding Griess reagent in culture medium (Welgene, Republic of Korea). In detail, 150 *μ*L of supernatant from each well was transferred to 96-well plate and then mixed with 150 *μ*L of Griess reagent solution. Mixtures were then incubated for 30 min at room temperature. OD was determined at 570 nm using a microplate reader.

### 2.7. RT-PCR

 Raw264.7 cell pellets were washed with ice-cold PBS, and RNAs were extracted using the easy-blue RNA extraction kit (Intron Biotech, Korea) according to the manufacturer's instructions. Total RNAs were quantified using NanoDrop ND-1000 spectrophotometer (NanoDrop Technologies Inc.) and subjected to reverse transcriptions using cDNA synthesis kit (TaKaRa, Japan). Conventional PCRs were then performed using appropriate primers. GAPDH was used as an internal control. Primer information was described in Table 1. 

### 2.8. IL-6 and TNF-*α* Measurement

IL-6 and TNF-*α* levels from Raw264.7 were measured by sandwich ELISA using BD Pharmingen mouse ELISA set. Cells in 6-well plates were treated with different concentrations of NG and incubated for 24 h. Cytokine levels were measured at 450 nm using a microplate reader.

### 2.9. Western Blot Analysis

Whole cell lysates were washed with ice-cold PBS and lysed with RIPA buffer. Equal amount of protein (30 *μ*g) was run on 10% SDS-PAGE and transferred to nitrocellulose membranes. Blotted membranes were then incubated overnight at 4°C with appropriate primary antibodies. After washing in PBS-tween 20 for 1 hour, membranes were incubated with appropriate HRP-conjugated secondary antibodies and bands were visualized with the enhanced chemiluminescence detection system (Amersham-Pharmacia Biotech, Buckinghamshire, UK).

### 2.10. Analyses of Cardiac Blood

Whole blood samples from mice were collected by cardiac punctures. Blood was loaded in Vacutainer tubes containing EDTA (BD Biosciences, USA). Analyses of WBC, neutrophil, and monocyte in blood were done using blood analyzer (Hemavet 950, Drew Scientific, Germany). 

### 2.11. Statistical Analysis

Data from experiments performed in triplicate presented mean ± standard deviation. Statistical significance was obtained by Student's *t*-test, and a *P* value less than 0.05 was considered statistically significant.

## 3. Results and Discussion

### 3.1. Water Extract of Deer Bone (NG) Induces RAW264.7 Cell Differentiation

**Figure 1:**
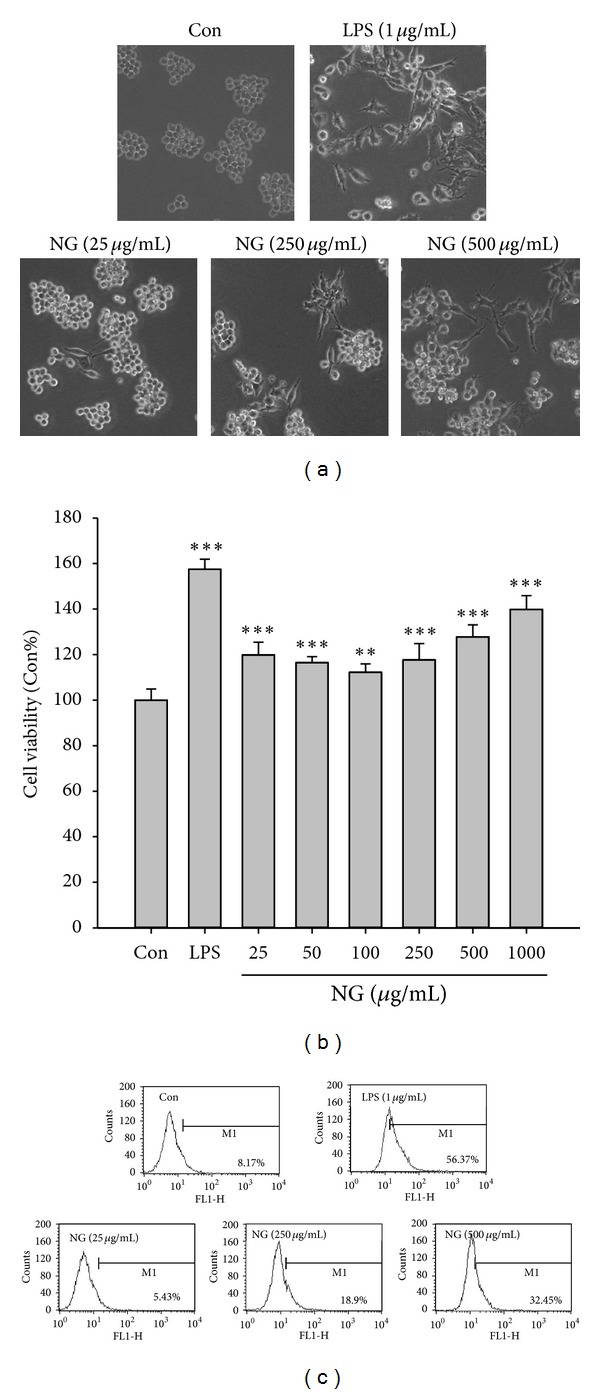
NG activates RAW264.7 cells. (a) Cells were seeded into 60 mm culture dish at a density of 3 × 10^5^ cells/dish. The next day, cells were treated with NG for 24 h. Cell surface was observed by taking a photograph using a camera attached to a microscope. (b) Cell viability was determined using WST assay after NG treatment. (c) ROS level was measured using DCFH-DA. Data are shown as the mean of three independent experiments (error bars are mean ± standard deviation (SD)) (*P < 0.05, **P < 0.01, and ***P < 0.001).

LPS has been known to induce a differentiation of monocytic Raw264.7 cells to macrophages. Consistently, LPS (1 *μ*g/mL) induced Raw264.7 differentiation in our experiment. Likewise, NG treatment also resulted in Raw264.7 cell differentiation in a dose-dependent manner (Figure 1(a)). In addition, both LPS and NG increased WST activities in RAW264.7 cells, indicating that NG induced activities of dehydrogenases (Figure 1(b)). Macrophages secrete ROS, when being activated [23]. So, we further measured ROS levels in Raw264.7 cells treated with different concentrations of NG or LPS. As seen in Figure 1(c), NG augmented ROS level in a dose-dependent manner. Thus, our data indicate that NG induces Raw264.7 cell differentiation to macrophages.

### 3.2. NG Increases NO Concentration and Induces Expression of iNOS and COX-2

**Figure 2:**
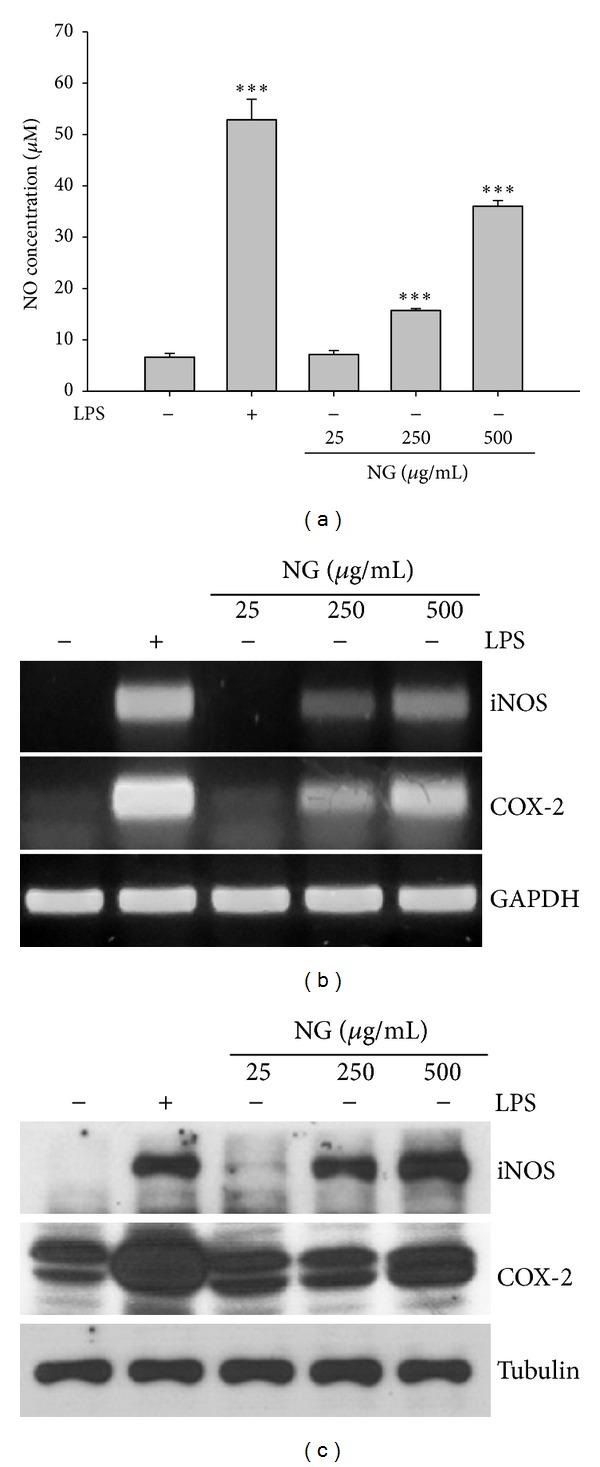
NG augmented NO concentration and iNOS and COX-2 expression. (a) NO production was measured using Griess reagent. (b) iNOS and COX-2 mRNA expression was measured by RT-PCR. (c) iNOS and COX-2 protein expression was measured by Western blot. Data are shown as the mean of three independent experiments (error bars are mean ± standard deviation (SD)) (*P < 0.05, **P < 0.01, and ***P < 0.001).

We next examined whether NG affects intracellular NO contents in Raw264.7 cells. When Raw264.7 cells were treated with different concentrations of NG or LPS as a positive control, both NG and LPS increased NO production (Figure 2(a)). As NO generation is associated with iNOS and COX-2, we further examined whether NG affects iNOS and COX-2. NG treatment increased expression of both iNOS and COX-2 (Figures 2(b) and 2(c)). 

### 3.3. NG Produces IL-6 and TNF-*α*

**Figure 3:**
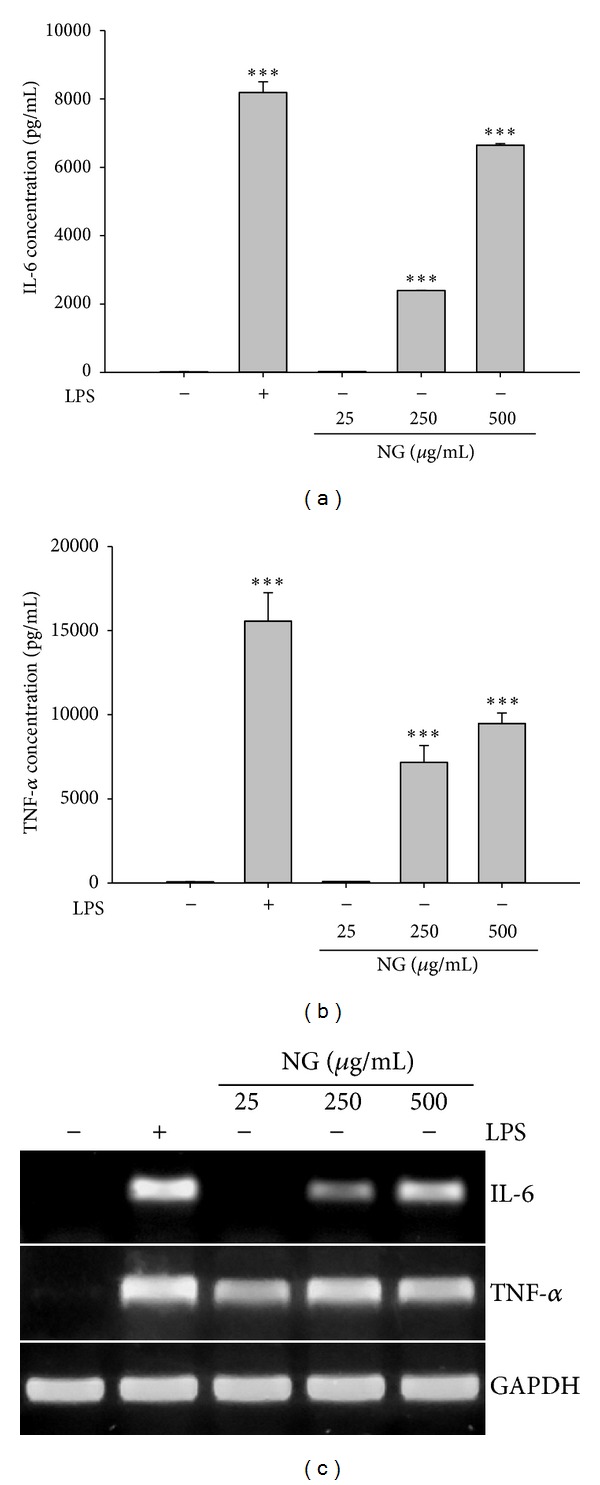
NG increased cytokines production. The release of IL-6 and TNF-*α* was measured by sandwich ELISA assay. Data are shown as the mean of three independent experiments (error bars are mean ± standard deviation (SD)) (*P < 0.05, **P < 0.01, and ***P < 0.001).

Macrophages react to microbial invasion through IL-6 and TNF-*α* secretion [17, 24]. Therefore, we measured the levels of IL-6 and TNF-*α* in Raw264.7 cells treated with different concentrations of NG for 24 hours. NG as well as LPS significantly increased levels of IL-6 and TNF-*α* (Figures 3(a) and 3(b)). This NG effect on IL-6 and TNF-*α* resulted from transcriptional regulation of both cytokines because NG treatment induced mRNA expression of either IL-6 or TNF-*α* (Figure 3(c)).

### 3.4. NG Induces Activation of MAPKs and NF-*κ*B

**Figure 4:**
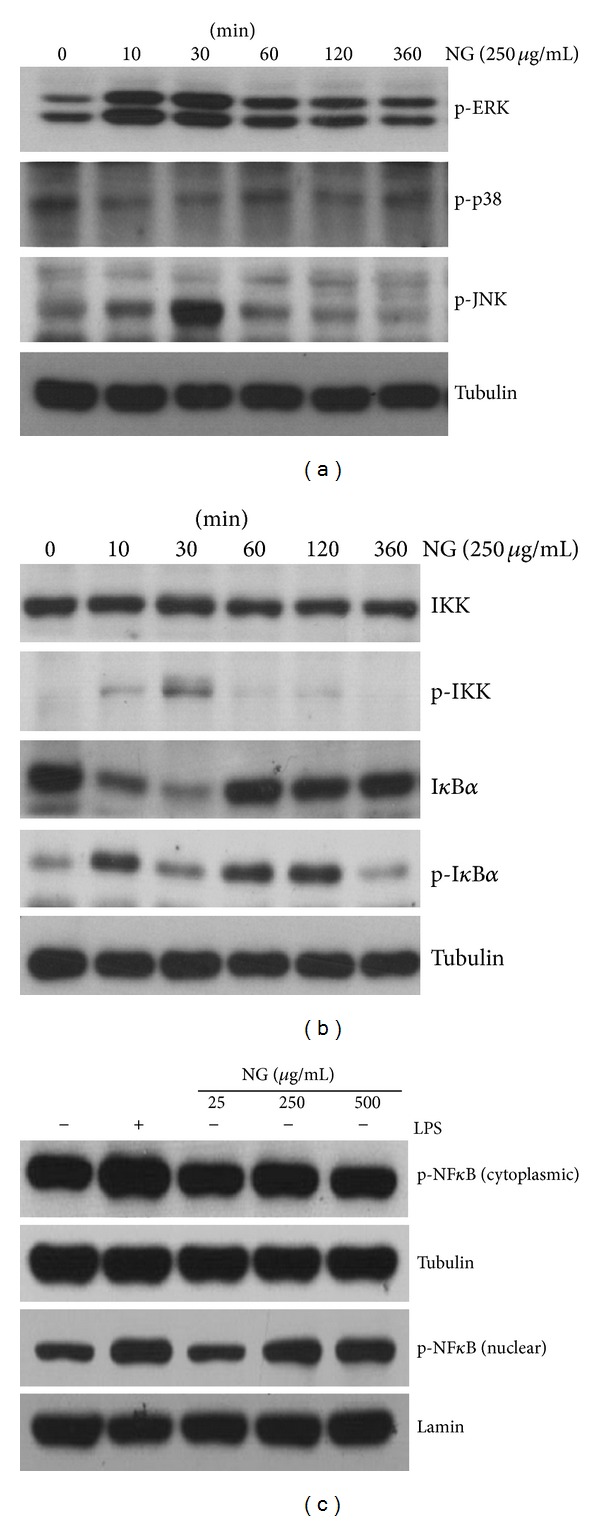
NG induced activation of MAPK and NF-*κ*B. (a) Whole-cell lysates were analyzed by Western blot for the detection of specific proteins, as indicated (p-ERK, p-p38, p-JNK, and tubulin). (b)Whole-cell lysates were analyzed by Western blot for the detection of specific proteins, as indicated (IKK, p-IKK, I*κ*B*α*, p-I*κ*B*α*, and tubulin). (c) Nuclear and cytosolic extracts were analyzed by Western blot for the detection of specific proteins, as indicated (p-NF-*κ*B, tubulin, and lamin).

**Figure 5:**
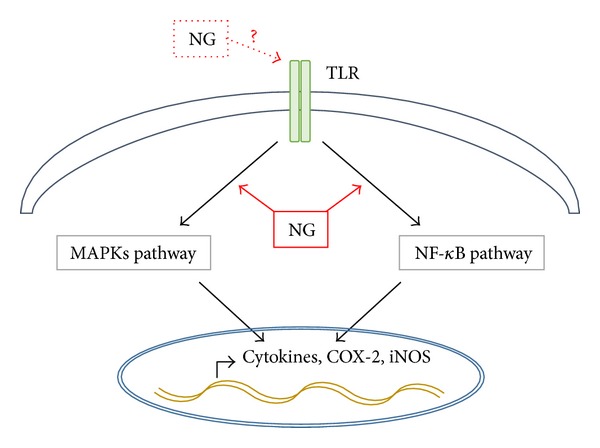
NG induces immune responses through MAPKs and NF-*κ*B activation (schematic diagram).

It has been reported that LPS activates macrophages through activation of MAPKs and NF-*κ*B [25]. So, we examined whether NG affects signaling pathways of MAPKs and NF-*κ*B in Raw264.7 cells. When Raw264.7 cells were treated with 250 *μ*g/mL of NG for 360 minutes, ERK phosphorylation was increased at 10 minutes after NG treatment and sustained until 30 minutes after NG treatment. JNK phosphorylation was increased at 10 minutes after NG treatment and peaked up at 30 minutes. However, NG appeared not to affect p38 phosphorylation (Figure 4(a)). We also found that NG activates NF-*κ*B pathway. IKK phosphorylation was gradually increased from 10 minutes and peaked up at 30 minutes after NG treatment. Likewise, I*κ*B phosphorylation was increased during that time points and total I*κ*B level was consistently reduced (Figure 4(b)). Accordingly, NG resulted in nuclear accumulation of phosphorylated form of NF-*κ*B, when phosphorylated form of NF-*κ*B in cytoplasmic and nuclear fractions were analyzed (Figure 4(c)). Thus, our data indicate that NG activates MAPK and NF-kB pathways as like LPS (Figure 5).

### 3.5. NG Alleviates in Mouse Neutropenia Model System

To verify NG effect in immune systems *in vivo*, we applied mouse neutropenia model system. In mice treated with NG, blood analyses presented a decrease of lymphocyte numbers and an increase of either neutrophils or eosinophils (Table 2). In NG-treated mice compared to normal mice, lymphocyte numbers were diminished by approximately 30% and neutrophil numbers were increased by approximately 30%. While numbers of monocytes and basophils were not altered significantly, eosinophil numbers were also significantly increased from 2.79% to 4.68%. Thus, our *in vivo* data indicate that NG treatment may improve immune defects including neutropenia. 

## 4. Conclusion

Our purpose of the study is to know whether NG has an immunomodulating effect. We confirmed that NG treatment increased ROS and promoted macrophagic differentiation of mouse monocytic Raw264.7 cells. In the *in vivo* study, we confirmed that NG improved immune defects including neutropenia. Hence, we conclude that NG is helpful for alleviating neutropenia.

## Figures and Tables

**Table 1:** The sequence of PCR primers.

Primer name	Sequences
IL-6	
Forward	5′-CAA GAG ACT TCC ATC CAG TTG C-3′
Reverse	5′-TTG CCG AGT TCT CAA AGT GAC-3′
TNF-*α*	
Forward	5′-ATG AGC ACA GAA AGC ATG ATC-3′
Reverse	5′-TAC AGG CTT GTC ACT GGA ATT-3′
COX-2	
Forward	5′-AAG ACT TGC CAG GCT GAA CT-3′
Reverse	5′-CTT CTG CAG TCC CAG GTT CAA-3′
iNOS	
Forward	5′-AAT GGC AAC ATC AGG TCG GCC ATC ACT-3′
Reverse	5′-GCT GTG TGT CAC AGA AGT CTC GAA CTC-3′
GAPDH	
Forward	5′-GAG GGG CCA TCC ACA GTC TTC-3′
Reverse	5′-CAT CAC CAT CTT CCA GGA GCG-3′

**Table:** (a)

	Normal	Negative Con	Positive Con	NG
WBC (K/uL)	10.89 ± 1.57	2.49 ± 1.48***	4.93 ± 0.71***	5.10 ± 1.48**
Lymphocytes (K/uL)	6.21 ± 1.27	0.76 ± 0.27***	1.77 ± 0.39**	2.00 ± 0.56**
Neutrophils (K/uL)	2.81 ± 0.77	1.07 ± 0.72*	2.75 ± 0.67	2.91 ± 0.79
Monocytes (K/uL)	0.59 ± 0.16	0.18 ± 0.07**	0.15 ± 0.07***	0.12 ± 0.04**
Eosinophils (K/uL)	0.22 ± 0.07	0.37 ± 0.06*	0.38 ± 0.06*	0.30 ± 0.13
Basophils (K/uL)	0.05 ± 0.02	0.04 ± 0.02	0.05 ± 0.02	0.07 ± 0.04

**Table:** (b)

	Normal	Negative Con	Positive Con	NG
Lymphocytes (%)	60.01 ± 6.85	41.93 ± 9.20*	41.64 ± 9.18*	31.05 ± 7.25***
Neutrophils (%)	29.87 ± 8.26	35.15 ± 19.12	55.95 ± 11.12**	60.31 ± 7.88***
Monocytes (%)	5.59 ± 0.64	5.21 ± 1.97	6.77 ± 1.62	4.06 ± 2.73
Eosinophils (%)	2.79 ± 1.33	8.99 ± 2.58**	7.12 ± 2.04**	4.68 ± 0.73*
Basophils (%)	0.50 ± 0.10	1.78 ± 0.89*	1.54 ± 0.23***	1.07 ± 0.62
